# COVID-19 and Diabetes Mellitus: From Pathophysiology to Clinical Management

**DOI:** 10.7759/cureus.31895

**Published:** 2022-11-25

**Authors:** Shahid S Memon, Dalia A Biswas

**Affiliations:** 1 Department of Anatomy, Jawaharlal Nehru Medical College, Datta Meghe Institute of Medical Sciences, Wardha, IND; 2 Department of Physiology, Jawaharlal Nehru Medical College, Datta Meghe Institute of Medical Sciences, Wardha, IND

**Keywords:** effects of covid-19 on diabetic patients, diabetes milletus in covid-19, covid-19, covid and diabetes, covid-19 and diabetes milletus

## Abstract

An increase in the severity of the coronavirus disease 2019 (COVID-19) was observed in patients infected with the acute severe metabolism syndrome coronavirus type 2 (SARS-CoV-2). Patients who have COVID-19 infection may also be more susceptible to hyperglycemia. When paired with other risk factors, hyperglycemia might alter immune and inflammatory responses, predisposing people to significant COVID-19 and perhaps deadly outcomes. Angiotensin-converting accelerator 2 (ACE2), a component of the renin-angiotensin-aldosterone system (RAAS), is the principal entry receptor for SARS-CoV-2; nevertheless, dipeptidyl enzyme 4 (DPP4) may potentially serve as a binding target. However, preliminary data did not indicate a substantial effect on the susceptibility to SARS-CoV-2 using glucose-lowering DPP4 inhibitors. Because of their pharmacologic characteristics, salt-glucose cotransporter 2 (SGLT2) inhibitors should not be advised for COVID-19 patients because they may have adverse effects. Currently, taking a hypoglycemic drug should be the most efficient way to manage acute glycemia. The majority of market proof is said to categorize two diabetes mellitus (DM) and fails to distinguish between the two primary categories of DM due to its widespread use. For grouping one DM and COVID-19, there is now some constrained proof available. Most of those findings are just preliminary, so further research will undoubtedly be required to determine the best course of action for DM patients.

## Introduction and background

The coronavirus disease 2019 (COVID-19), which is brought on by the unusual coronavirus known as SARS-CoV-2, was initially identified in China in the lunar calendar month of 2019, and it has since spread over the entire world. According to the COVID-19 dashboard of the World Health Organization, as of October 29, 2020, 44,351,506 globally verified COVID-19 cases and 1,171,255 deaths had been reported [1]. Calculations show that the COVID-19 fatality rate ranges from 0.5% to 1%. COVID-19 was formally responsible for 905,235 (79%) of the 1,22,300 more all-effects fatalities that happened in the USA between 1 March and 30 May 2020. It should be highlighted that COVID-19 mortality and yearly respiratory disease death are not equal because deaths from both diseases do not duplicate frontline clinical situations in the same way. For instance, towns afflicted by the COVID-19 epidemic have a very difficult time getting supplies like ventilators and ICU facilities [2].

According to calculations, the COVID-19 mortality rate fluctuates between 0.5% and 1%. Of the 122,300 more all-cause fatalities that occurred in the USA between 1 March and 30 May 2020, COVID-19 was formally accountable for 905,235 (79%) of them. Because deaths from both diseases do not replicate frontline clinical circumstances in the same way, it should be made clear that COVID-19 fatality and yearly respiratory problems mortality are not equivalent. For instance, it is extremely difficult for towns affected by the COVID-19 pandemic to obtain resources like ventilators and ICU facilities [3]. Symptoms of COVID-19 usually appear in patients five to six days after first becoming infected. Equivalent to SARS-CoV and the Middle East respiratory syndrome (MERS)-CoV, SARS-CoV-2 infection typically presents with mild symptoms for the first two weeks but has the potential to worsen and result in shock, multi-organ involvement, severe inflammation, and acute respiratory distress syndrome (ARDS) [4]. Patients who are at risk of developing severe COVID-19 or passing away have a variety of risk factors, such as advanced age and male gender, as well as underlying health conditions such as cardiovascular diseases (CVDs), obesity, and/or type one or type two diabetes mellitus (T1DM or T2DM) [5]. Numerous preliminary studies have shown that underpinning CVDs and diabetes mellitus are common in COVID-19 patients admitted to intensive care units. T2DM is frequently an illness of advanced age, hence it is still unknown if DM may be a COVID-19 risk factor over and above old age [6].

There has been a review of the basic and clinical evidence regarding the potential interactions between DM and COVID-19. However, data in this area is sporadic with numerous articles [7]. This review covers the most recent developments in DM and COVID-19 and shifts the primary emphasis to treatment advice for DM patients at risk for or with COVID-19-related illnesses. Most existing research does not differentiate between different types of diabetes and is mostly focused on T2DM due to its high prevalence [8].

## Review

Increased COVID-19 severity

There is a correlation between severe COVID-19 and diabetes, according to two early case series of critically ill COVID-19 patients admitted to ICUs in the USA. The prevalence of diabetes was reported to be 58% and 33%, respectively [9]. Numerous processes are believed to be responsible for the chronicity of COVID-19 being more pronounced in diabetics. As already mentioned, people with polygenic disease diabetes mellitus have a higher chance of developing thromboembolic consequences and impairment to vital organs due to factors such as protein synthesis, glucotoxicity, and epithelial tissue damage from inflammatory, aerophilic stress [10]. Additionally, a drug commonly used in the therapeutic treatment of COVID-19 patients, such as generic corticosteroids or antiviral medications, may aggravate hyperglycemia [11].

COVID-19 and T1DM

Due to the high incidence of this form of polygenic disease diabetes mellitus, evidence about the influence of diabetes on COVID-19 typically has not distinguished between the major types and is typically related to T2DM. As noted in this section, certain crucial information is provided specifically for T1DM [12].

T1DM newly discovered

Case reports have distinguished between patients with newly diagnosed T1DM who had acidosis at the outset of COVID-19 and those with newly diagnosed T1DM who did not develop acidosis until many weeks after they appeared to be recovering from COVID-19 [13]. These results raise the issue of whether this biochemical disorder will be induced by SARS-CoV-2. One study indicated that, while receiving therapy for COVID-19, 29 patients, some of whom had a normal HbA1c level upon admission but who were not previously diagnosed with diabetes, acquired hyperglycemia [14]. The number of pediatric T1DM patients admitted to specialized Italian polygenic disease centers was lower than anticipated. In contrast, specialized hospitals in northwest London, UK, observed more patients than anticipated presenting with severe acidosis, indicating a potential rise in the number of patients with new-onset T1DM [15]. These inconsistent results could be explained by the small number of patients examined, or they could be the result of changes in the availability of medical care during the COVID-19 pandemic [16]. There was no variation from the anticipated number of newly identified pediatric T1DM patients, according to a society research from the Federal Republic of Germany. But a comparable study discovered a statistically significant rise in diabetic acidosis and serious acidosis in children and adolescents with newly diagnosed T1DM [17]. This discovery may indicate individuals wanting to postpone hospital admission due to their concern over contracting SARS-CoV-2 infection, according to one likely explanation for the data. If a true connection between COVID-19 and fresh T1DM exists, it will become more obvious as the COVID-19 pandemic spreads and more people are researched [18].

Metabolic control in T1DM outpatients

Many groups first from Italian Republic, Europe, and Britain have discovered that people with T1DM and without COVID-19 have shown no degradations in glycemic control during the pandemic compared to an effective level before the pandemic, and occasionally even show improvements up to the mark [19]. Self-reported regular exercise was found to be decreased during internment, and similar patterns of nutritional consumption and sleep were also discovered. These findings may duplicate the settings below, where glycemic control is easier to achieve. This effect might be problematic because of the limited access to food, drugs, and glucose in underdeveloped nations. Look at the strips and the medical services [20]. Due to facilities of online consultation and telemedicine spurred on by the COVID-19 pandemic, developed countries' glycemic treatment practices may have been influenced [21].

T1DM patients that are hospitalized and have COVID-19

According to a population-based investigation from a European nation, those with T1DM have a similar risk of hospitalization to those without it (0.21% against 0.17%). Hospitalized T1DM patients treated for COVID-19 in this research and another in the USA shared similar metabolic traits with T1DM patients. WHO were admitted to hospitals for other medical reasons, and their HbA1c values weren't greater than those of COVID-19 patients [22]. T1DM patients that are hospitalized and have COVID-19. According to a population-based investigation from a European nation, those with T1DM have a similar risk of hospitalization to those without it (0.21% against 0.17%). Hospitalized T1DM patients treated for COVID-19 in this research and another in the USA shared similar metabolic traits with T1DM patients. WHO were admitted to hospitals for other medical reasons, and their HbA1c values weren't greater than those of COVID-19 patients [23].

T1DM and COVID-19 outcomes

Two population-based assessments first from the United Kingdom showed that T1DM patients had a higher rate of death than people without the disease. Older, with accumulating HbA1c levels, blood vessel high blood pressure, a urinary organ functional impairment, and prior vast events, and patients with T1DM who were in explicit danger for these conditions (myocardial infarct, stroke, or heart failure) [24]. To prevent thromboembolic occurrences and their complications during the COVID-19 pandemic, we tend to suggest that doctors should think about writing prescriptions for antiplatelet or anticoagulating agents to patients with diabetes more actively. This is because clients with polygenic disease diabetes mellitus have an increased risk of developing thromboembolism [25].

The precise cellular and molecular causes of the elevated blood coagulability in COVID-19 individuals are still unknown, and the standard bar does not always work when thromboembolism is present [26]. But for patients with severe COVID-19 who were at high thromboembolic risk, such as those with elevated D-dimer levels, low mass Heparin therapy improved outcomes [27]. Therefore, it would be wise to start pharmaceutical medical care in hospitalized patients with mild to severe COVID-19, even though it wouldn't be necessary for those with a modest course of the illness [28].

Even while there is no evidence to indicate any immediate benefits of GLP1 analog on the risk of occlusion, numerous animal studies have found that therapy with these drugs delays the growth of fat and settles down plaques in vascular arteries and arterial blood vessel arches [29]. In vitro injection of GLP1 decreased the expression of the matrix metalloproteinases pair and MCP1, as well as the translocation of NF-B-p65, which is associated with a higher risk of thromboembolism. A vast outcome study found that using a long GLP1 analog to treat patients with T2DM decreased the risk of stroke. Additionally, diabetic people might benefit from selecting anti-diabetic drugs that may be able to lower the chance of thromboembolic events [30].

Potential drug interactions

**Figure 1:**
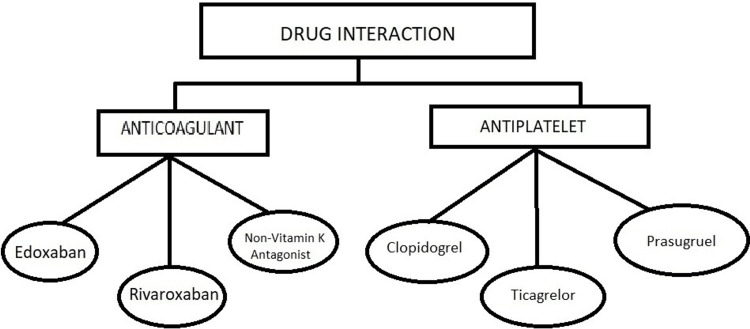
COVID-19 Oral Drugs COVID-19: Coronavirus disease 2019

Investigational COVID-19 drugs and commonly used oral anticoagulants or antiplatelet medications have the potential to interact. In some nations, including China and India, a combination drug of lopinavir and protease inhibitor, 2 proteinase inhibitors, is used for treating COVID-19 patients. The hemoprotein P450 3A4 (CYP3A4) enzyme metabolism is inhibited by these proteolytic enzyme inhibitors, which results in lower concentrations of the antiplatelet drug clopidogrel's active ingredient [31]. In contrast, by preventing the metabolism of ticagrelor, these proteinase inhibitors may enhance the antiplatelet effects of ticagrelor. Anti-hemorrhagic factor antagonist medications like apixaban and betrixaban require dosage adjustments since they may interact negatively with proteinase inhibitors. On the contrary, lopinavir and protease inhibitors frequently enhance the therapeutic effects of edoxaban and rivaroxaban, non-vitamin K antagonist oral anticoagulants, discouraging concurrent treatment with these medications [32]. As a result, caution should be exercised when prescribing drugs that may influence CYP3A4 activity since they may alter the effects of antiplatelet medicines or anticoagulants that are processed by the CYP3A4 system. Remdesivir has beneficial effects in reducing the amount of time needed for recovery in very adults hospitalized with COVID-19, according to an early study. It may be an ester analog of a polymer-dependent RNA enzyme. Remdesivir was discovered to have no significant interactions with anticoagulants or antiplatelet medications. Investigational COVID-19 treatments and canal anticoagulants generally do not show any significant medication interactions [33] (Figure *1*).

Antiviral therapies

Because it inhibits the transmembrane enzyme amino acid pair (TMPRSS2), which promotes the pathogen's entry into the host cell, camostat mesylate may be an amino acid under investigation for its capacity to prevent infectious agent entry [34]. We investigated whether SARS-CoV-2 infection could lead to cell depletion in insulin-producing pancreatic cells in order to relate COVID-19 to diabetes mechanistically. We discovered that the ACE2 entrance factor, the SARS-CoV-2 receptor, and associated entry factors (TMPRSS2, NRP1, and TRFC) are all expressed in cells, with NRP1 being specifically expressed at a high level. We found that SARS-CoV-2 primarily infects human islet cells in vitro and infects human pancreatic cells in individuals who died from COVID-19. We showed that SARS-CoV-2 infection reduces sugar levels and secretion from the pancreas and causes cell death, each of which is prevented by NRP1 suppression [35]. By boosting hypoglycemic agent sensitivity and elevating duct gland function, anti-inflammatory also has a glucose-lowering impact, which has allowed it to be given as an antidiabetic medicine medication in some countries [36].

Antiviral drugs like lopinavir might worsen pre-existing diabetes mellitus (DM), increase the risk of hyperglycemia and new polygenic disease diabetes mellitus, increase the likelihood of diabetic ketoacidosis, and prolong the risk of both conditions. These drugs have up to 50% lessened cell function and hypoglycemic agent sensitivity in HIV individuals [37]. Pharmacologic interactions with the concurrent administration of glucose-lowering medications are another problem with enzyme inhibitors. For instance, substance P (neuromodulator) operates as an associate CYP3A4/5 inhibitor, boosting plasma levels of the DPP4i saxagliptin, and as an inducer of uridine 5′-diphospho-glucuronosyltransferase, reducing levels of the SGLT2i canagliflozin. Therefore, patients taking these medicine combinations are advised to regularly monitor their blood sugar levels and adjust their dosage [38]. Remdesivir, an ester analog of a polymer-dependent RNA enzyme, reduced endotoxemia, liver disease, and hypoglycemic agent sensitivity in mice given a high-fat diet. In contrast, in RCTs including social groups and Chinese patients, the rise in blood levels of aldohexose was comparable in between remdesivir-treated and placebo-treated groups. Therefore, more research is necessary to determine how it affects the metabolism of aldohexose.

## Conclusions

Patients with diabetes should be aware that the COVID-19 pandemic will raise blood levels of aldohexose. As a result, they must adhere to the clinical guidelines for the management of diabetes very closely, as outlined here. We provide the following general advice for patients and healthcare professionals: Patients should be more vigilant about their compliance with prescribed medications, including hypoglycemic agent injections, as well as their levels in the blood of aldohexose, which should be checked more frequently than previously. Patients should see their doctor if aldohexose blood concentrations are consistently above normal. Healthcare providers must place a lot of emphasis on good food consumption and physical exercise in patients suffering from DM in light of current global quarantine regulations. Patients should be advised to visit their doctor immediately if they have symptoms such as a persistent cough, excessive liquid body material production, fever, or a sudden spike in aldohexose levels.

Furthermore, it is strongly advised that patients closely follow their doctor's prescriptions and avoid statements spread through various media outlets, including the internet, as they frequently cannot withstand scientific scrutiny. Most importantly, to decrease the likelihood of infection in patients with DM, all healthcare providers and their patients should closely adhere to common precautions including social seclusion, wearing a mask, washing hands, and improperly using disinfectants. Telehealth or virtual consultations could help reduce the risk involved in face-to-face interactions among patients and medical professionals. These extra measures may help lessen the risk of SARS-CoV-2 transmission while providing the general public with safe and effective care.

Coronavirus infections are well known to have a significant impact on the management of DM because they exacerbate inflammation and modify physiological responses, making it difficult to control blood sugar levels. Additionally, SARS-CoV-2 infection raises the risk of occlusion and is more likely to cause metabolic failure in DM patients than in those without DM. It should be kept in mind, nevertheless, that Decadron's effectiveness in treating COVID-19 has been thoroughly tested in well-designed RCTs like the RECOVERY study, whereas no compelling RCTs are conducted for anti-inflammatory.

In conclusion, the COVID-19 global pandemic poses serious health risks, especially for people with diabetes. It has not yet been possible to develop a COVID-19 vaccine or effective treatment. Therefore, the easiest solution is to avoid infection in the first place. Under these conditions, people with diabetes should make a concerted effort to maintain good health and reduce potential risk factors. A significant area for present and future research is the best management approach for these individuals, including the choice of glucose-lowering, medicine, and lipid-lowering drugs.
